# Cardioprotective Effects of Resistance Training on Obesity

**DOI:** 10.5935/abc.20190085

**Published:** 2019-05

**Authors:** Marcelo Diarcadia Mariano Cezar, Luana Urbano Pagan, Ricardo Luiz Damatto, Aline Lima, Mariana Janini Gomes

**Affiliations:** 1 Faculdade de Ciências Sociais e Agrárias de Itapeva (FAIT), Itapeva, SP - Brazil; 2 Faculdade de Medicina de Botucatu, Universidade Estadual Paulista (UNESP), Botucatu, SP - Brazil

**Keywords:** Obesity/complications, Cardiovascular Diseases/mortality, Diabetes Mellitus, Hypertension, Mice, Diet, High-fat, Exercise, Quality of Life

Obesity is characterized as a complex metabolic disorder and is associated with several complications such as cardiovascular diseases, diabetes, renal dysfunction, liver dysfunction and cancer, resulting in impairment of quality of life.^1^

The pathogenesis of obesity has a multifactorial origin and the oxidative stress may play an important role. Studies on animals and cell culture have reported how oxidative stress can contribute to the development of obesity, causing increased preadipocytes proliferation, adipocyte differentiation, and size of mature adipocytes, resulting in increased production of pro-inflammatory cytokines such as tumor necrosis factor alpha (TNF-α).^2,3^

Experimental model of high fat diet-induced obesity aims to reproduce the characteristics observed in human, such as the development of cardiovascular abnormalities.^4,5^ The study of Effting et al.^6^ evaluated the effects of resistance training on parameters of oxidative stress and inflammation in mice with high fat diet-induced obesity.

Currently, regular physical exercise has been recommended for the treatment of obesity. Exercise practicing results in numerous health benefits, such as improvement in body composition, physical capacity, insulin resistance, endothelial function, arterial hypertension, antioxidant status and quality of life.^7,8^

Data presented by the authors of the article "Resistance Exercise Modulates Oxidative Stress Parameters and TNF-α Content in the Heart of Mice with Diet-Induced Obesity" showed important cardioprotective effects of resistance training, which resulted in decreased levels of lipid peroxidation and reactive oxygen species, modulation of antioxidant enzymes activity and a decrease in myocardial TNF-α concentration of obese mice^6^. Similarly, Alves et al.^9^ observed that eight weeks of resistance exercise was associated with an improvement on inflammatory profile in the heart of rats with myocardial infarction.

The effects of resistance exercise on oxidative stress have been investigated mainly in skeletal muscle.^10-12^ There are few studies evaluating the effects of resistance exercise on the redox status of the cardiac muscle in the literature. Therefore, Effting et al.^6^ presented relevant data supporting resistance exercise as a therapeutic approach to obesity, being able to prevent or mitigate metabolic changes and improve the quality of life.
